# Intravascular Lithotripsy in Calcified Coronary Lesions: A Single-Center Experience in “Real-World” Patients

**DOI:** 10.3389/fcvm.2022.829117

**Published:** 2022-02-21

**Authors:** Angelo Mastrangelo, Giovanni Monizzi, Stefano Galli, Luca Grancini, Cristina Ferrari, Paolo Olivares, Mattia Chiesa, Giuseppe Calligaris, Franco Fabbiocchi, Piero Montorsi, Antonio L. Bartorelli

**Affiliations:** ^1^Department of Interventional Cardiology, Centro Cardiologico Monzino, Istituto di Ricovero e Cura a Carattere Scientifico (IRCCS), University of Milan, Milan, Italy; ^2^Bioinformatics and Artificial Intelligence Facility, Centro Cardiologico Monzino, Istituto di Ricovero e Cura a Carattere Scientifico (IRCCS), University of Milan, Milan, Italy; ^3^Department of Electronics, Information and Biomedical Engineering, Politecnico di Milano, Milan, Italy; ^4^Department of Clinical Sciences and Community Health, Cardiovascular Section, University of Milan, Milan, Italy; ^5^Department of Biomedical and Clinical Sciences “Luigi Sacco”, University of Milan, Milan, Italy

**Keywords:** coronary lesions, calcium, balloon angioplasty, lithotripsy, drug-eluting stents

## Abstract

**Objectives:**

This study aims to describe the outcome of intravascular lithotripsy (IVL) when used with different indications and to assess the short- and long-term outcomes of IVL-facilitated percutaneous coronary intervention (PCI).

**Background:**

Intravascular lithotripsy can improve the results of PCI of calcified coronary lesions with a low rate of periprocedural complications.

**Methods:**

A total of 105 consecutive patients with 110 calcified lesions underwent IVL. A total of 87 *de novo* lesions were treated by IVL with the following indications: 25 before attempting other balloon-based devices (primary IVL), 51 after the failure of non-compliant balloon dilatation (secondary IVL), and 11 after stent implantation because of stent under expansion (bailout IVL). In 23 lesions, IVL was used for the treatment of in-stent restenosis (ISR). Effectiveness (angiographic success) and safety [major adverse cardiovascular events (MACEs) and IVL-related procedural complications] endpoints were assessed.

**Results:**

Angiographic success was achieved in 84.6% of lesions. Early MACEs were periprocedural MI only, ranging from 6.7 to 20% depending on MI definition. The flow-limiting dissections rate was 2.7%. A total of five (4.5%) IVL balloons ruptured during treatment with subsequent vessel perforation in 1 case. MACEs at 12 months were 13.3%, with TLR occurring in 8 lesions (12% primary IVL, 0% secondary IVL, 0% bailout IVL, and 21.7% IVL for ISR, *p* = 0.002).

**Conclusion:**

Treatment of calcified coronary lesions with IVL in a “real-world” setting can be performed with high success, low rate of procedural complications, and an acceptable MACEs rate. Target lesion failure may be more frequent when IVL is performed for the treatment of ISR due to calcium-mediated stent under expansion.

## Introduction

Up to 30% of patients undergoing percutaneous coronary intervention (PCI) have calcified lesions (1). Heavy calcification still represents a major challenge for successful PCI. Mortality, major adverse cardiovascular events (MACEs), target vessel failure, and stent thrombosis (ST) occur more frequently as the result of calcium-mediated poor lesion preparation, stent under expansion, and stent malapposition (2).

Several technological developments have been introduced to aid in the treatment of severely calcified coronary lesions. Recently, intravascular lithotripsy (IVL), a balloon-based calcium-modifying technique, has been introduced as a promising strategy, which is simple to use, with a high rate of procedural success and a low rate of complications (3–6). Nevertheless, data regarding IVL use in distinctive anatomical settings and different clinical indications are scarce and long-term data using this therapeutic approach are lacking. The aim of this study was to evaluate the outcome of IVL-facilitated PCI in an all-comers population with calcified coronary lesions, focusing on the short- and long-term results related to different uses of IVL in the “real-world” practice.

## Materials and Methods

### Study Population

This retrospective, observational study included consecutive patients with calcified coronary lesions treated with IVL from December 31, 2018 to December 31, 2020 at the Centro Cardiologico Monzino, University of Milan, Italy. During the study period, 4,191 patients with 7,540 lesions underwent PCI, and 105 (2.5%) patients with 110 (1.5%) calcified coronary lesions were treated with IVL. Medical records and coronary angiograms of these patients were examined. All the patients provided informed consent according to local institutional practice.

### Definitions

Coronary angiograms were assessed by two experienced operators. Lesion calcification was visually stratified into moderate or severe using a previously described angiographic method based on the use of readily apparent densities noted within the vascular wall at the stenosis level. Moderate lesion calcification was defined as radiopaque densities noted during the cardiac cycle involving only one side of the vascular wall, whereas severe lesion calcification was defined as radiopaque densities noted without cardiac motion before contrast injection generally involving both sides of the arterial wall (7). Based on the symmetry of coronary artery narrowing, lesions were angiographically classified as eccentric or concentric. Eccentric lesions were defined as stenotic lesions that had one of its luminal edges in the outer one-quarter of the apparent normal vessel lumen, whereas concentric lesions were defined using the same criteria while involving both luminal edges. Whenever possible, multiple angiographic angles were used to confirm the lesion classification (8).

### Devices and Procedures

Treatment with IVL of *de novo* calcified lesions and in-stent restenosis (ISR) due to calcium-mediated under expansion of a previously implanted stent was performed using the Shockwave C^2^ balloon-based coronary system (Shockwave Medical Inc., Santa Clara, CA, USA), according to the Instructions for Use. An extension catheter was used to deliver the IVL balloon to the target lesion, if needed. Compliant, non-compliant, and/or cutting balloons, but not atherectomy, were used at discretion of the operator at any time during the intervention. Three different IVL approaches were identified for the treatment of *de novo* coronary lesions:

- Primary IVL (P-IVL), before attempting other conventional balloon-based devices (semicompliant, non-compliant, and/or cutting balloons). After IVL, further predilatation with non-compliant balloons was performed before stent implantation at the discretion of the operator;
- Secondary IVL (S-IVL), after suboptimal predilatation with conventional balloon-based devices;
- Bailout IVL (B-IVL), after stent implantation, as a rescue strategy to correct calcium-mediated acute stent under expansion refractory to high-pressure balloon inflation.

In patients with ISR, IVL was used only after failure of high-pressure stent dilatation with a non-compliant balloon performed during the index or a previous procedure (ISR–IVL group). The decision on the strategy and timing of IVL use was made by the operator at the time of the intervention.

*De-novo* lesions were treated with drug-eluting stent (DES) implantation, whereas ISR was treated by either DES implantation, drug-coated balloon (DCB), or balloon-only angioplasty, at discretion of the operator. Final lesion postdilatation with a non-compliant balloon was performed to optimize the angiographic result, aiming at <20% residual stenosis by visual estimate.

All the patients received guidelines-based antithrombotic therapy depending on the clinical presentation, procedure complexity, and ischemic and bleeding risk (9).

### Angiographic Analysis

Coronary angiography was performed according to local standards. Quantitative coronary angiography (QCA) was performed offline with a dedicated software (CAAS 8.2 Workstation, Pie Medical Imaging, Maastricht, The Netherlands). Several angiographic indexes were derived: reference vessel diameter (RVD), lesion length, minimal lumen diameter (MLD), percent diameter stenosis (%DS), and acute gain (postprocedure MLD minus preprocedure MLD). Lesions were classified according to the modified American College of Cardiology-American Heart Association guidelines (10). Long stenoses were defined as > 15 mm lesions.

### Endpoints

The primary effectiveness endpoint was angiographic success, defined as the composite of successful IVL balloon delivery to the target lesions, adequate stent expansion, residual stenosis <20%, and final thrombolysis in myocardial infarction (TIMI) 3 flow. A residual stenosis <50% was included for the secondary effectiveness endpoint. The primary safety endpoint was the occurrence of MACEs, defined as the composite of cardiac death, myocardial infarction (MI), and target vessel revascularization (TVR) during follow-up. Early (within 30 days from the index procedure) and late (after 30 days from the index procedure) events were assessed. Three definitions of periprocedural MI were used: (1) creatinine kinase (CK)-based definition [post-PCI CK-MB peak > 3x the upper limit of normal (ULN)], (2) Fourth Universal Definition, and (3) The Society for Cardiac Angiography and Interventions (SCAI) definition (11, 12). Spontaneous MI during follow-up was assessed using the contemporary definition and considered target vessel MI (TVMI) when appropriate (11). Target vessel revascularization, target lesion revascularization (TLR), and ST were defined according to the Academic Research Consortium-2 Consensus Document (13). The secondary safety endpoint was freedom from IVL-related serious complications [flow-limiting coronary dissection (types D to F), persistent slow-flow/no-reflow phenomena, acute ST, coronary perforation, malignant arrhythmias (ventricular tachycardia or ventricular fibrillation), and IVL device failure (malfunction or burst of the balloon)].

### Follow-Up

Follow-up data were collected using hospital records and telephone interviews.

### Statistical Analysis

Statistical analysis was performed using Stata/SE 12.0. Continuous data were summarized as mean ± SD or median [interquartile range (IQR)]. Categorical data are presented as numbers with percentages. The primary endpoint (angiographic success) was based on QCA results. We performed paired *t-*test for comparison of QCA results at baseline and after PCI for the overall cohort and each treatment group. Analysis between the four groups was performed using the chi-squared test, Fisher's exact test, ANOVA, or the Kruskal–Wallis test, as appropriate. We also performed a subgroup analysis of patients who met or did not meet the primary effectiveness endpoint (Supplementary Tables 1–6) and of those with and without TLR (Supplementary Tables 7–12) using the chi-squared test, Fisher's exact test, unpaired Student's *t-*test or Mann–Whitney *U*-test, as appropriate. The logistic regression analysis was used to identify TLR predictors. Procedural and periprocedural variables significantly associated with TLR at univariate analysis were assessed. Differences were considered significant at *p* < 0.05.

## Results

**Figure 1:**
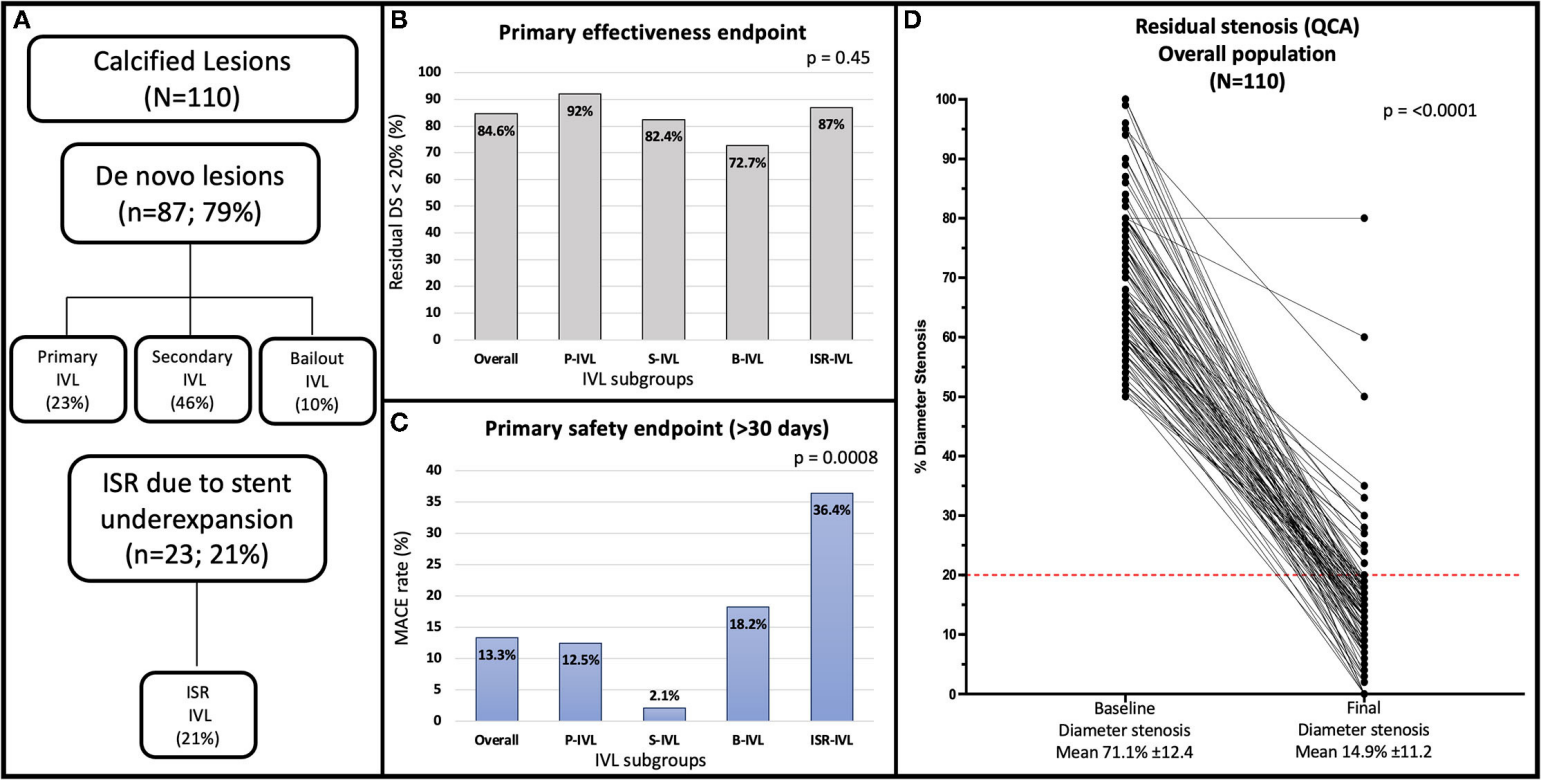
**(A)** Flowchart of the study. **(B)** Angiographic success rate in the overall population and treatment subgroups. **(C)** Late (median 12.9 months) MACE rate in the overall population and treatment subgroups. **(D)** Quantitative coronary angiography showing percent diameter stenosis at baseline and after the procedure for the overall population. The red line indicates the <20% residual stenosis threshold after procedure included in the primary effectiveness endpoint of the study. B-IVL, bailout intravascular lithotripsy; ISR, in-stent restenosis; ISR-IVL, intravascular lithotripsy for in-stent restenosis; IVL, intravascular lithotripsy; MACE, major adverse cardiovascular event; P-IVL, primary intravascular lithotripsy; QCA, quantitative coronary angiography; S-IVL, secondary intravascular lithotripsy.

The study flow chart and main findings are shown in Figure 1.

### Patients and Procedures

Patient baseline characteristics are shown in Table 1. Lesion and procedural characteristics are summarized in Tables 2 and 3, respectively. A total of one-hundred thirteen IVL catheters were successfully delivered to the target lesions. Three coronary lesions were treated with two IVL catheters (one lesion with significant vessel tapering, one with suboptimal lesion preparation after complete IVL treatment, and one following balloon burst after 20 pulses before adequate lesion expansion). To improve backup support, 7F guide catheters and guide catheter extensions were used in 7.3 and 27.3% of the lesions, respectively. Overall, a mean of 64 ± 21 pulses was delivered. A median of 3 balloons (IQR 2–4) per lesion other than the IVL balloon was used for predilatation. One balloon (IQR 1–2) was used in the P-IVL group, 3 balloons (IQR 3–4) in the S-IVL group, 2 balloons (IQR 1–4) in the B-IVL group, and 3 balloons (IQR 2–5) in the ISR–IVL group. Before IVL, predilatation with non-compliant, high-pressure balloons was performed in 63.6% of the lesions [0% in P-IVL, 100% in S-IVL, 54.6% in B-IVL, and 56.5% in ISR-IVL (a previous PCI with conventional technique failed to dilate the stent in the remaining 43.5% of ISR-IVL)], while adjunctive plaque modification with a cutting balloon was performed in 6.4% of lesions (1 in S-IVL and 6 in ISR-IVL). After IVL and before stenting, high-pressure balloon dilatation was performed in 59.1% of lesions (36% in P-IVL, 70.6% in S-IVL, and 87% in ISR-IVL). In the B-IVL group, IVL was used after stenting as a rescue strategy to correct stent under expansion despite high-pressure postdilatation with non-compliant balloons. Overall, ninety-one (82.7%) lesions were treated with DES implantation (100% in P-IVL, 96.1% in S-IVL, 100% in B-IVL, and 26.1% in ISR-IVL), while DCB was used in 12 (10.9%) lesions, all in the ISR-IVL group. Final, high-pressure balloon postdilatation was performed in 76.4% of the lesions (92% in P-IVL, 82.4% in S-IVL, 90.9% in B-IVL, and 39.1% in ISR-IVL cases).

**Table 1:** Baseline clinical characteristics of overall population and patient subgroups.

	**Overall**	**Primary IVL**	**Secondary IVL**	**Bailout IVL**	**ISR IVL**
No. of patients	105	24	48	11	22
Male, *n* (%)	89 (84.8)	20 (83.3)	42 (87.5)	9 (81.8)	18 (81.8)
Age (mean ± SD)	71.4 ± 7.6	70.9 ± 6.7	70.6 ± 8.0	73.6 ± 8.7	72.45 ± 7.19
**Risk factors**					
Obesity^*^, *n* (%)	16 (15.2)	4 (16.7)	6 (12.5)	0 (0)	6 (27.3)
Hypertension, *n* (%)	78 (74.3)	19 (79.2)	34 (70.8)	7 (63.6)	18 (81.8)
Hypercholesterolemia, *n* (%)	79 (75.2)	19 (79.2)	35 (72.9)	8 (72.7)	17 (77.3)
Smoking, *n* (%)	52 (49.5)	13 (54.2)	20 (41.7)	5 (45.5)	14 (63.6)
Current, *n* (%)	13 (12.4)	4 (16.7)	4 (8.3)	2 (18.2)	3 (13.6)
Former, *n* (%)	39 (37.1)	9 (37.5)	16 (33.3)	2 (18.2)	3 (13.6)
Family history of CVD, *n* (%)	30 (28.6)	6 (25.0)	16 (33.3)	4 (36.4)	4 (18.2)
Diabetes mellitus, *n* (%)	29 (27.6)	9 (37.5)	11 (22.9)	2 (18.2)	7 (31.8)
History of CAD, *n* (%)	65 (61.9)	13 (54.2)	26 (54.2)	5 (54.5)	21 (95.5)
Prior MI, *n* (%)	33 (31.4)	6 (25.0)	15 (31.3)	3 (27.3)	9 (40.9)
Prior CABG, *n* (%)	17 (16.2)	4 (16.7)	9 (18.8)	2 (18.2)	2 (9.1)
Prior PCI, *n* (%)	52 (49.5)	12 (50.0)	15 (31.3)	3 (27.3)	22 (100.0)
LVEF (mean ± SD)	57 ± 8	56 ± 9	57 ± 8	62 ± 6	58 ± 9
Prior stroke, *n* (%)	5 (4.8)	1 (4.2)	3 (6.3)	1 (9.1)	0 (0)
Chronic kidney disease,^†^ *n* (%)	26 (24.8)	5 (20.8)	8 (16.7)	6 (54.6)	7 (31.8)
Creatinine (mean ± SD), mg/dl	1.07 ± 0.43	1.01 ± 0.22	1.04 ± 0.34	1.08 ± 0.43	1.08 ± 0.43
End-stage renal disease, *n* (%)	1 (0.95)	0 (0)	0 (0)	1 (9.1)	0 (0)
Peripheral artery disease, *n* (%)	28 (26.7)	5 (20.8)	15 (31.3)	2 (18.2)	6 (27.3)
**Clinical Presentation**					
Chronic coronary syndrome, *n* (%)	93 (88.6)	24 (100)	43 (89.6)	11 (100)	15 (68.2)
Angina, *n* (%)	43 (40.9)	10 (41.7)	18 (37.5)	5 (45.5)	10 (45.5)
CCS I, *n* (%)	4 (3.8)	0 (0)	2 (4.2)	5 (10.4)	5 (10.4)
CCS II, *n* (%)	18 (17.1)	5 (20.8)	8 (16.7)	3 (27.3)	2 (9.1)
CCS III, *n* (%)	16 (15.2)	4 (16.7)	5 (10.4)	2 (18.2)	5 (22.7)
CCS IV, *n* (%)	5 (4.8)	1 (4.17)	3 (6.3)	0 (0)	1 (4.6)
Dyspnoea, *n* (%)	20 (19.1)	3 (12.5)	10 (20.8)	4 (36.4)	3 (16.7)
Acute coronary syndrome, *n* (%)	12 (11.4)	0 (0)	5 (10.4)	0 (0)	7 (31.8)
Unstable angina, *n* (%)	1 (0.95)	0 (0)	0 (0)	0 (0)	1 (4.6)
NSTEMI, *n* (%)	9 (8.6)	0 (0)	5 (10.4)	0 (0)	4 (18.2)
STEMI, *n* (%)	2 (1.9)	0 (0)	0 (0)	0 (0)	2 (9.1)

*Values are mean ± SD or n (%)*.

*CABG, coronary artery bypass grafting; CAD, coronary artery disease; CCS, Canadian Cardiovascular Society; CVD, cardiovascular disease; ISR, in-stent restenosis; IVL, intravascular lithotripsy; LVEF, left ventricular ejection fraction; MI, myocardial infarction; NSTEMI, non-ST-segment elevation MI; PCI, percutaneous coronary intervention*.

* *Defined as body mass index > 30 kg/m^2^*.

† *Defined as glomerular filtration rate < 60 ml/min/1.73 m^2^*.

**Table 2:** Lesions and procedural characteristics of the overall population and patient subgroups.

	**Overall**	**Primary IVL**	**Secondary IVL**	**Bailout IVL**	**ISR IVL**
No. of IVL treated lesions	110	25	51	11	23
**CAD type**					
One-vessel CAD, *n* (%)	26 (23.6)	6 (24)	12 (23.5)	2 (18.2)	6 (26.1)
Two-vessel CAD, *n* (%)	43 (39.1)	11 (44)	20 (39.2)	3 (27.3)	9 (39.1)
Three-vessel CAD, *n* (%)	41 (37.3)	8 (32)	19 (37.3)	6 (54.6)	8 (34.8)
LM disease, ^*^n (%)	22 (20)	7 (28)	11 (21.6)	2 (18.2)	2 (8.7)
**No. of treated vessels during index-procedure**					
One-vessel PCI, *n* (%)	59 (53.6)	13 (52)	28 (54.9)	5 (45.5)	13 (56.5)
Two-vessels PCI, *n* (%)	44 (40)	10 (40)	21 (41.2)	5 (45.5)	8 (34.8)
Three-vessels PCI, *n* (%)	7 (6.4)	2 (8)	2 (3.9)	1 (9.1)	2 (8.7)
**IVL-treated vessel**					
LM, *n* (%)	11 (10)	5 (20)	4 (7.8)	1 (9.1)	1 (4.4)
LAD, *n* (%)	56 (50.9)	12 (48.0)	26 (51)	6 (54.6)	12 (52.2)
CX, *n* (%)	14 (12.7)	1 (4)	9 (17.7)	2 (18.2)	2 (8.7)
RCA, *n* (%)	29 (26.4)	7 (28)	12 (23.5)	2 (18.2)	8 (34.8)
**Lesion location**					
Ostial, *n* (%)	16 (14.5)	2 (8)	11 (21.6)	1 (9.1)	2 (8.7)
Proximal, *n* (%)	54 (49.1)	11 (44)	24 (47.1)	5 (45.5)	14 (60.9)
Medial, *n* (%)	28 (25.5)	8 (32)	11 (21.6)	3 (27.3)	6 (26.1)
Distal, *n* (%)	12 (10.9)	4 (16)	5 (9.8)	2 (18.2)	1 (4.4)
**Lesion characteristics**					
Type B1,^†^ *n* (%)	2 (1.8)	0 (0)	2 (3.9)	0 (0)	0 (0)
Type B2,^†^ *n* (%)	38 (34.6)	12 (48)	20 (39.2)	6 (54.6)	0 (0)
Type C,^†^ *n* (%)	47 (42.7)	13 (52)	29 (56.9)	5 (45.5)	0 (0)
ISR, *n* (%)	23 (20.9)	0 (0)	0 (0)	0 (0)	23 (100)
Stent thrombosis, *n* (%)	1 (0.9)	0 (0)	0 (0)	0 (0)	1 (4.4)
Bifurcation lesions, *n* (%)	21 (19.1)	10 (40)	8 (15.7)	1 (9.1)	2 (8.7)
CTO, *n* (%)	2 (1.8)	0 (0)	1 (2)	1 (9.1)	0 (0)
**Coronary calcification**					
Moderate, *n* (%)	16 (14.5)	1 (4)	2 (3.9)	2 (18.2)	11 (47.8)
Severe, *n* (%)	94 (85.5)	24 (96)	49 (96.1)	9 (81.8)	12 (52.2)
**Lesion characteristics**					
Eccentric, *n* (%)	82 (74.6)	22 (88)	39 (76.5)	9 (81.8)	12 (52.2)
Concentric, *n* (%)	28 (25.5)	2 (12)	12 (23.5)	2 (18.2)	11 (47.8)
**Lesion access**					
Severe tortuosity, *n* (%)	19 (17.3)	2 (8)	12 (23.5)	2 (18.2)	3 (13)
**Procedural characteristics**					
Radial artery, *n* (%)	86 (78.2)	19 (76.0)	39 (76.5)	9 (81.9)	19 (82.6)
Femoral artery, *n* (%)	30 (27.2)	7 (28.8)	15 (29.4)	2 (18.2)	6 (26.1)
7F catheter, *n* (%)	8 (7.3)	2 (8.0)	3 (5.9)	1 (9.1)	2 (8.7)
Guide catheter extension, *n* (%)	30 (27.3)	7 (28)	16 (31.4)	3 (27.3)	4 (17.4)
Protected PCI, *n* (%)	4 (3.6)	1 (4.0)	2 (3.9)	0 (0)	1 (4.4)
IVUS guided, *n* (%)	26 (23.6)	8 (32)	9 (17.7)	3 (27.3)	6 (26.1)
OCT guided, *n* (%)	12 (10.9)	4 (16)	3 (5.9)	0 (0)	5 (21.7)
Contrast agent (mean ± SD), mL	265 ± 130	301 ± 136	259 ± 117	264 ± 135	239 ± 149
Fluoroscopy time (mean ± SD), min	24.08 ± 13.99	25.85 ± 8.56	24.44 ± 17.34	25.35 ± 12.67	20.76 ± 10.82
Total DAP (mean ± SD), CGy × cm^2^	14,205 ± 8,949	14,367 ± 6,881	14,760 ± 10,719	15,152 ± 7,768	12,344 ± 7,192

*Values are mean ± SD, or n (%)*.

*DAP, dose area product; CAD, coronary artery disease; CX, circumflex; CTO, chronic total occlusion; FFR, fractional flow reserve; ISR, in-stent restenosis; IVL, intravascular lithotripsy; IVUS, intravascular ultrasound; LAD, left anterior descending; LM, left main; OCT, optical coherence tomography; PCI, percutaneous coronary intervention; RCA, right coronary artery*.

* *Defined as diameter stenosis > 50%*.

† *American College of Cardiology-American Heart Association classification*.

**Table 3:** Procedural characteristics of lesion treatment in the overall population and patient subgroups.

	**Overall**	**Primary IVL**	**Secondary IVL**	**Bailout IVL**	**ISR IVL**
No. of IVL treated lesions	110	25	51	11	23
**IVL**					
No. of IVL catheters delivered, n	113	25	53	12	23
Lesions treated with 1 IVL catheter, *n* (%)	107 (97.3)	25 (100)	49 (96.1)	10 (90.9)	23 (100)
Lesions treated with 2 IVL catheters, *n* (%)	3 (2.7)	0 (0)	2 (3.9)	1 (9.1)	0 (0)
Number of pulses (mean ± SD), *n*	64 ± 21	65 ± 20	61 ± 23	68 ± 11	67 ± 22
Diameter of IVL balloon, mm	3.0 ± 0.4	3.1 ± 0.5	2.9 ± 0.4	2.9 ± 0.3	3.2 ± 0.4
**Predilatation**					
Pre-IVL high-pressure dilatation, *n* (%)	70 (63.6)	0 (0)	51 (100)	6 (54.6)	13 (56.5)
Largest balloon diameter (mean ± SD), mm	2.9 ± 0.4	–	2.8 ± 0.4	2.8 ± 0.3	3.0 ± 0.4
Mean pressure (mean ± SD), atm	19 ± 4	–	19 ± 4	19 ± 5	19 ± 5
Pre-IVL cutting balloon, *n* (%)	7 (6.4)	0 (0)	1 (2)	0 (0)	6 (26.1)
Largest balloon diameter (mean ± SD), mm	2.9 ± 0.4	–	3.0	–	2.9 ± 0.4
Post-IVL high-pressure dilatation, *n* (%)	65 (59.1)	9 (36)	36 (70.6)	–	20 (87)
Largest balloon diameter (mean ± SD), mm	3.1 ± 0.5	3.2 ± 0.4	2.9 ± 0.3	–	3.4 ± 0.5
Mean pressure (mean ± SD), atm	20 ± 8	16 ± 3	19 ± 6	–	25 ± 10
Median of balloons/lesion (IQR), n	3 (2–4)	1 (1–2)	3 (3–4)	2 (1–4)	3 (2–5)
**Lesion treatment**					
DES implantation, *n* (%)	91 (82.7)	25 (100)	49 (96.1)	11 (100)	6 (26.1)
Median of DES/lesion	1 (1;2)	1 (1;2)	2 (1;3)	1 (1;3)	0 (0;1)
0 DES, *n* (%)	19 (17.3)	0 (0)	2 (3.9)	0 (0)	17 (73.9)
1 DES, *n* (%)	46 (41.8)	14 (56)	23 (45.1)	6 (54.5)	3 (13)
2 DES, *n* (%)	23 (20.9)	5 (20)	13 (25.5)	2 (18.2)	3 (13)
3 DES, *n* (%)	20 (18.2)	6 (24)	11 (21.6)	3 (27.3)	0 (0)
4 DES, *n* (%)	2 (3.9)	0 (0)	2 (3.9)	0 (0)	0 (0)
Stented length (mean ± SD), mm	35.5 ± 20.5	40.8 ± 25.9	34.6 ± 18.6	34.9 ± 17.2	22.3 ± 7.7
DCB used, *n* (%)	12 (10.9)	0 (0)	0 (0)	0 (0)	12 (52.2)
Median of DCB/lesion	0 (0;0)	–	–	–	1 (0;1)
0 DCB, *n* (%)	98 (89.1)	–	–	–	11 (47.8)
1 DCB, *n* (%)	9 (8.2)	–	–	–	9 (39.1)
2 DCB, *n* (%)	3 (2.7)	–	–	–	3 (13)
**Postdilatation**					
High-pressure dilatation before IVL, *n* (%)	11 (10)	–	–	11 (100)	–
Largest balloon diameter (mean ± SD), mm	3.1 ± 0.4	–	–	3.1 ± 0.4	–
Mean pressure (mean ± SD), atm	22 ± 7	–	–	22 ± 7	–
Final high-pressure dilatation, *n* (%)	84 (76.4)	23 (92)	42 (82.4)	10 (90.9)	9 (39.1)
Largest balloon diameter (mean ± SD), mm	3.6 ± 0.6	3.8 ± 0.7	3.6 ± 0.6	3.3 ± 0.2	3.5 ± 0.6
Mean pressure (mean ± SD), atm	19 ± 5	17 ± 3	19 ± 4	21 ± 5	24 ± 9

*Values are n (%), mean ± SD, or median (IQR)*.

*DCB, drug-coated balloon; DES, drug-eluting stent; ISR, in-stent restenosis; IVL, intravascular lithotripsy; IQR, interquartile range*.

### Angiographic Assessment

Results of QCA in the entire population and patient subgroups are shown in Table 4, Figure 1, and Supplementary Figure 1. Overall, mean RVD was 3.26 ± 0.63 mm, mean lesion length 20.46 ± 15.99 mm, and 56 (50.9%) lesions were long. At baseline, the average MLD was 0.98 ± 0.46 mm and final MLD 2.77 ± 0.62 mm (*p* < 0.0001). Average DS at baseline and post PCI were 70.1 ± 12.4 and 14.9 ± 11.2%, respectively. On average, acute diameter gain was 1.79 ± 0.6 mm.

**Table 4:** Quantitative coronary angiography of treated lesions in the overall population and patient subgroups.

	**Overall**	**Primary IVL**	**Secondary IVL**	**Bailout IVL**	**ISR IVL**	***p***
No. of IVL treated lesions	110	25	51	11	23	
**Reference vessel**						
Diameter (mean ± SD), mm	3.26 ± 0.63	3.58 ± 0.6	3.22 ± 0.64	3.04 ± 0.47	3.13 ± 0.63	0.03
No. of lesions with RVD >3 mm, *n* (%)	70 (63.6)	20 (80)	30 (58.8)	7 (63.6)	13 (56.5)	0.27
**Lesion—basal assessment**						
Length (mean ± SD), mm	20.46 ± 15.99	26.21 ± 22.29	18.96 ± 12.64	24.65 ± 18.22	15.54 ± 11.56	0.08
No. of long lesions, ^*^n (%)	56 (50.9)	13 (52)	26 (51)	8 (72.7)	9 (39.1)	0.34
MLD (mean ± SD), mm	0.98 ± 0.46	1.07 ± 0.51	0.97 ± 0.4	0.84 ± 0.39	0.96 ± 0.56	0.56
DS (mean ± SD), %	70.1 ± 12.39	70.36 ± 12.84	69.33 ± 11.75	73.18 ± 10.30	70.04 ± 14.56	0.83
**Lesion—final assessment**						
MLD (mean ± SD), mm	2.77 ± 0.62	3.08 ± 0.53	2.72 ± 0.65	2.50 ± 0.64	2.69 ± 0.57	0.03
DS (mean ± SD), %	14.94 ± 11.2	13.8 ± 5.53	15.27 ± 13.35	17.64 ± 16.8	14.13 ± 7	0.79
Acute Gain, mm	1.79 ± 0.6	2.01 ± 0.55	1.74 ± 0.64	1.66 ± 0.52	1.73 ± 0.60	0.23

*Values are mean ± SD, n (%)*.

*DS, diameter stenosis; ISR, in-stent restenosis; IVL, intravascular lithotripsy; MLD, minimal lumen diameter*.

* *Defined as lesion length > 15 mm*.

### Study Endpoints

The primary effectiveness endpoint (residual DS < 20%) was achieved in 88 (83.8%) patients and 93 (84.6%) lesions. A residual in-stent DS < 50% was achieved in 102 (97.1%) patients and 107 (97.3%) lesions. The IVL catheter could not be delivered in 1 (0.9%) lesion of a tortuous right coronary artery. The result of IVL was judged unsatisfactory to proceed with stent implantation in 1 (0.9%) chronic total occlusion of the right coronary artery. Adequate stent expansion occurred in 107 (97.3%) lesions (100% in P-IVL, 96.1% in S-IVL, 90.9% in B-IVL, 100% in ISR-IVL; *p* = 0.41). Table 5 summarizes the composite and individual components of the effectiveness endpoints of the treatment subgroups.

**Table 5:** Effectiveness endpoints in the overall population and patient subgroups.

	**Overall**	**Primary IVL**	**Secondary IVL**	**Bailout IVL**	**ISR IVL**	***p***
No. of IVL treated lesions	**110**	**25**	**51**	**11**	**23**	
IVL device delivery and treatment	109 (99.1)	25 (100)	50 (98)	11 (100)	23 (100)	0.99
DES/DCB successful expansion	107 (97.3)	25 (100)	49 (96.1)	10 (90.9)	23 (100)	0.41
Final TIMI 3 flow	110 (100)	25 (100)	51 (100)	11 (100)	23 (100)	–
Residual DS < 20%	93 (84.6)	23 (92)	42 (82.4)	8 (72.7)	20 (87)	0.45
Residual DS < 50%	107 (97.3)	25 (100)	49 (96.1)	10 (90.9)	23 (100)	0.41

*Values are n (%)*.

*DS, diameter stenosis; ISR, in-stent restenosis; IVL, intravascular lithotripsy; TIMI, Thrombolysis in Myocardial Infarction; DES, drug-eluting stent; DCB, drug-coated balloon*.

At 30 days, no cardiac death, TLR, TVR nor spontaneous MI occurred and the primary safety endpoint of MACEs was driven solely by periprocedural MI, whose rate was dependent on the definition used, ranging from 6.7% (Fourth Universal and SCAI definitions) to 20% (CK-MB-based definition). The rate of periprocedural MI in each treatment group is detailed in Table 6.

**Table 6:** Safety endpoints in the overall population and patient subgroups.

	**Overall**	**Primary IVL**	**Secondary IVL**	**Bailout IVL**	**ISR IVL**	***p***
No. of patients	105	24	48	11	22	
**Primary safety endpoint**						
Median follow-up (months)	12.3 (8.6–19.7)	9.5 (8.1–15.1)	16.8 (9.7–21.3)	8.9 (7.8–11.4)	12.1 (8.6–19.2)	0.006
**In-hospital MACE**						
Cardiac death, *n* (%)	0 (0)	0 (0)	0 (0)	0 (0)	0 (0)	-
Peri-procedural MI (CK-MB >3 × URL), *n* (%)	21 (20)	3 (12.5)	9 (18.8)	2 (18.2)	7 (31.8)	0.42
Peri-procedural MI (IV UD), *n* (%)	7 (6.7)	2 (8.3)	2 (4.2)	2 (18.2)	1 (4.5)	0.38
Peri-procedural MI (SCAI definition), *n* (%)	7 (6.7)	1 (4.2)	3 (6.3)	1 (9.1)	2 (9.1)	0.9
Stent thrombosis,^*^n (%)	0 (0)	0 (0)	0 (0)	0 (0)	0 (0)	-
Target lesion revascularization,^*^n (%)	0 (0)	0 (0)	0 (0)	0 (0)	0 (0)	-
Target vessel revascularization,^*^n (%)	0 (0)	0 (0)	0 (0)	0 (0)	0 (0)	-
**Early MACE (<30 days)**	0 (0)	0 (0)	0 (0)	0 (0)	0 (0)	-
Cardiac death, *n* (%)	0 (0)	0 (0)	0 (0)	0 (0)	0 (0)	-
Spontaneous MI	0 (0)	0 (0)	0 (0)	0 (0)	0 (0)	-
Stent thrombosis,^*^n (%)	0 (0)	0 (0)	0 (0)	0 (0)	0 (0)	-
Target lesion revascularization,^*^n (%)	0 (0)	0 (0)	0 (0)	0 (0)	0 (0)	-
Target vessel revascularization,^*^n (%)	0 (0)	0 (0)	0 (0)	0 (0)	0 (0)	-
**Late MACE (>30 days)**	14 (13.3)	3 (12.5)	1 (2.1)	2 (18.2)	8 (36.4)	0.0008
Cardiac death, *n* (%)	2 (1.9)	0 (0)	1 (2.1)	0 (0)	1 (4.5)	0.58
Spontaneous MI (TVMI), *n* (%)	5 (4.8)	0 (0)	0 (0)	1 (9.1)	4 (18.2)	0.003
Stent thrombosis,^*^n (%)	2 (1.8)	0 (0)	0 (0)	0 (0)	2 (8.7)	0.051
Probable, *n* (%)	1 (0.9)	0 (0)	0 (0)	0 (0)	1 (4.3)	0.31
Definite, *n* (%)	1 (0.9)	0 (0)	0 (0)	0 (0)	1 (4.3)	0.31
Target vessel revascularization,^*^n (%)	13 (11.8)	3 (12)	0 (0)	2 (18.2)	8 (34.8)	<0.0001
Target lesion revascularization,^*^n (%)	8 (7.3)	3 (12)	0 (0)	0 (0)	5 (21.7)	0.002
Target vessel non-target lesion revascularization,^*^n (%)	7 (6.4)	1 (4)	0 (0)	2 (18.2)	4 (17.4)	0.004
**Secondary safety endpoint**						
Freedom from procedural complications, *n* (%)	97 (92.4)	20 (83.3)	45 (93.8)	10 (90.9)	22 (100)	0.15
Serious angiographic complications,^*^n (%)	4 (3.6)	2 (8)	1 (1.9)	1 (9.1)	0 (0)	0.21
Flow-limiting dissection,^*^n (%)	3 (2.7)	2 (8)	0 (0)	1 (9.1)	0 (0)	0.07
Perforation,^*^n (%)	1 (0.9)	0 (0)	1 (1.9)	0 (0)	0 (0)	0.99
Acute stent thrombosis,^*^n (%)	0 (0)	0 (0)	0 (0)	0 (0)	0 (0)	-
Persistent slow-flow or no-reflow phenomena, ^*^n (%)	0 (0)	0 (0)	0 (0)	0 (0)	0 (0)	-
Serious IVL-related arrhythmias,^*^n (%)	0 (0)	0 (0)	0 (0)	0 (0)	0 (0)	-
Ventricular tachycardia,^*^n (%)	0 (0)	0 (0)	0 (0)	0 (0)	0 (0)	-
Ventricular fibrillation,^*^n (%)	0 (0)	0 (0)	0 (0)	0 (0)	0 (0)	-
Failure of the IVL system,^*^n (%)	5 (4.6)	2 (8)	3 (5.9)	0 (0)	0 (0)	0.69
Malfunction,^*^n (%)	0 (0)	0 (0)	0 (0)	0 (0)	0 (0)	-
Balloon burst,^*^n (%)	5 (4.6)	2 (8)	3 (5.9)	0 (0)	0 (0)	0.69

*Values are n (%)*.

*CK-MB, creatine kinase muscle and brain; ISR, in-stent restenosis; IVL, intravascular lithotripsy; MI, myocardial infarction; SCAI, Society for Cardiovascular Angiography and Intervention; TLR, target lesion revascularization; TVR, target vessel revascularization; TVMI, target vessel myocardial infarction; URL, upper reference limit; IV UD, Fourth Universal Definition*.

* *Calculations were based on a per lesion basis (overall n = 110, primary IVL therapy n = 25, secondary IVL therapy n = 51, bailout IVL therapy n = 11, ISR IVL therapy n = 23)*.

Long-term (>30 days from the index procedure) follow-up was obtained in all the patients at a median of 12.3 (8.6–19.7) months. Minimum follow-up was 6 months, except for one patient who died 3.9 months after the index procedure for non-cardiac causes. A total of ninety-one (86.7%) patients with 96 (87.3%) lesions were MACEs-free at long-term follow-up. The rate of long-term MACEs was 12.5% in P-IVL, 2.1% in S-IVL, 18.2% in B-IVL, and 36.4% in ISR-IVL; *p* < 0.001). A total of three (2.9%) patients died during follow-up. One death (0.95%) occurred at 3.9 months (metastatic colorectal cancer), one (0.95%) at 13.9 months (heart failure in wild-type cardiac amyloidosis), and one (0.95%) at 15.7 months (sudden cardiac death), 8 days after TLR for ISR (probable ST). Late events were mostly TVR, performed in 13 (12.4%) patients and 13 (11.8%) lesions. Seven (6.4%) were TVR and 8 (7.3%) TLR. Primary PCI was performed in 5 (4.8%) patients with 5 (4.8%) TVMI. In 3 (2.7%), the culprit lesion was not at the target site, while in 2 (1.8%) it was at the target site. One (0.95%) of patients with MI was in the ISR–IVL group and had definite ST. The rate of late events in the different groups is reported in Table 6.

All TLR were clinically driven and were because of the ISR (12% in P-IVL, 0% in S-IVL, 0% in B-IVL, 21.7% in ISR-IVL; *p* = 0.002). Factors associated with ISR are listed in Supplementary Tables 7–12. Table 7 details relevant periprocedural factors related to ISR and TLR. Lithotripsy-related complications occurred in 8 (7.6%) patients with 9 (8.2%) lesions. There were 3 (2.7%) flow-limiting dissections, 2 in the P-IVL group (1 type D and 1 type F), and 1 in the B-IVL group (type F). Five (4.5%) IVL-balloon bursts occurred, 2 in the P-IVL group, and 3 in the S-IVL group. One of the balloon bursts in the S-IVL group was complicated by vessel perforation (Ellis Type III), successfully treated with a covered stent. Periprocedural MI occurred in 1 of the 3 IVL-related dissections and in 2 of the 5 IVL-balloon bursts, regardless of the definition used. Acute ST, persistent slow-flow/no-flow phenomena, and serious arrhythmic events did not occur (Table 6).

**Table 7:** Multivariate procedural and periprocedural predictors of TLR by the logistic regression analysis.

**Predictors**	***Beta***	***p***
ISR (related to calcium-mediated stent under expansion)	3.33	0.006
High-pressure predilatation before IVL therapy	−2.12	0.04
IVL-related procedural complications^*^	2.73	0.07
Peri-procedural MI (IV UD definition)	2.62	0.08
Periprocedural MI (SCAI definition)	−0.03	0.98

*Values are n*.

*ISR, in-stent restenosis; IVL, intravascular lithotripsy; MI, myocardial infarction; SCAI, Society for Cardiovascular Angiography and Intervention; IV UD, Fourth Universal Definition*.

* *Procedural complications include flow-limiting coronary dissections, the burst of the IVL balloon, and coronary perforation*.

### Antiplatelet and Anticoagulation Therapy

A total of 97 (92.4%) patients were treated with antiplatelet drugs, while 8 (7.6%) who were on oral anticoagulation at the time of PCI received triple therapy with aspirin and clopidogrel for 1 to 6 months and dual therapy with anticoagulant and aspirin or clopidogrel thereafter (Supplementary Table 13).

## Discussion

Intravascular lithotripsy is a new and effective tool in the armamentarium of interventional cardiology for the treatment of complex coronary calcified lesions. Compared with atherectomy devices, IVL is more user-friendly and has a shorter learning curve thanks to the balloon-based technology. This study describes the results of coronary IVL in an all-comer population with moderately or severely calcified coronary lesions treated in a single, high-volume PCI center in Italy.

### Main Findings

The key findings of our study are as follows:

- Intravascular lithotripsy was feasible and effective. Angiographic success was high and independent of clinical presentation, lesion type (*de novo* or ISR), and lithotripsy indication;
- Intravascular lithotripsy was safe. Overall, the periprocedural MI rate was higher than previously reported, but with no relevant clinical consequences. Device-related complications were rare and no serious early events were observed. The late MACEs rate was acceptable, and was higher (mainly TVR) in the ISR-IVL group.

### Feasibility and Effectiveness Endpoints

Our study suggests that IVL can be easily and effectively performed regardless of coronary lesion complexity. Indeed, it was not possible to treat only one lesion in a tortuous right coronary artery because of the failed delivery of the IVL catheter. The need for larger diameter (7 French) guiding catheters and guiding catheter extensions for backup support (Tables 2, 3) was low demonstrating the trackability of the IVL balloon catheter. However, its crossing profile range (0.044–0.047″), higher than that (0.015–0.018″) of non-compliant coronary balloons in the market, often required lesion predilatation, similarly to the pivotal Disrupt CAD III trial (3). A residual DS < 20% with TIMI 3 coronary flow was achieved in 93 (84.6%) lesions of 88 (83.8%) patients, whereas a residual DS < 50% was obtained in 107 (97.3%) lesions of 102 (97.1%) patients (Table 5). Angiographic success proved to be similar regardless of lesion type (*de novo* or ISR) and treatment indication (*p* = 0.45; Table 5). Moreover, IVL indication did not affect acute diameter gain (*p* = 0.23; Table 4). These results confirm previous reports suggesting that high-angiographic success can be achieved with IVL in both *de novo* and ISR related to underexpanded stents (3–6, 14). Compared with the previous experiences, most of the lesions in our study were angiographically eccentric (Table 2). Initially, IVL was predominantly used in concentric calcific lesions because of its calcium specificity. Nevertheless, recently new data on the use of IVL in eccentric lesions suggested no significant differences in terms of procedural and clinical outcomes when compared with concentric lesions (8, 15). Our experience confirms these results.

As anticipated, *de novo* lesions were treated with IVL using a primary (without previous lesion predilatation), a secondary (after suboptimal lesion preparation with conventional, high-pressure balloon dilatation), or a bailout (after stent implantation with evidence of calcium-mediated “acute” stent under expansion) approach. In the absence of an evidence-based indication for the use of IVL as an upfront or a secondary technique (3), the procedural strategy was deliberately chosen by the operator. In our analysis, patients in the P-IVL and S-IVL presented similar clinical, lesions, and procedural characteristics. Notably, lesions did not differ in terms of calcification severity, lesion eccentricity, and lesion complexity, according to Ellis classification (Table 2). Thus, it is difficult to identify those factors influencing the choice of a primary instead of a secondary IVL strategy. Considering the higher number of lesions in the S-IVL group, it is possible that in the “real-world” setting operators prefer to initially adopt a conventional technology (non-compliant, scoring, and cutting balloons) in order to reduce the costs of the procedure. Indeed, when good lesions preparation is achieved, this strategy allows avoiding the use of more expensive devices (such as IVL). From our experience, it is difficult to propose one approach instead of the other since the procedural and clinical results were similar in the two treatment groups. It is interesting to note that the number of balloons needed for predilatation was significantly lower in P-IVL (1, IQR 1–2) compared with S-IVL (3, IQR 3–4; *p* = 0.02). In addition, a lower number of lesions in P-IVL underwent high-pressure balloon dilatation after IVL and before stenting (Table 3, *p* = 0.006). It will be interesting to verify in future studies with a larger patient population if this finding translates into a reduction of procedure duration, radiation exposure, complications, and cost when IVL is used as a primary instead of a secondary indication and lesions present similar anatomical characteristics.

Regarding acute stent under expansion, a condition associated with poor acute (ST) and long-term (ISR and late ST) outcome (16, 17), IVL can achieve adequate stent expansion despite a non-significant trend toward higher residual stenosis compared with the other treatment indications (Table 5). One of the causes of stent under expansion is deep calcification. In this scenario, plaque-modifying devices relying on tissue compression or debulking might fail to overcome the restraint of the calcified plaque, while IVL may be more effective by inducing multiple calcium cracks (18). However, acute stent under expansion should be avoided by identifying those lesions requiring enhanced preparation. An in-depth assessment by intravascular imaging (IVUS or OCT) may be useful for this purpose (19), although these imaging tools are expensive and time-consuming, facts that are still limiting their widespread clinical use. Furthermore, urgent stent implantation may be required, particularly when aggressive predilatation of heavily calcified lesions causes complications, such as occlusive dissection or vessel perforation. Therefore, it is reassuring to know that IVL can be effective even as a bailout strategy when high-pressure balloon dilatation fails to achieve optimal stent expansion.

### Early Major Adverse Cardiovascular Event

The primary safety endpoint of the study was the MACEs rate within 30 days. Early events were exclusively procedure-related MI. Several periprocedural MI definitions have been previously used in IVL studies. In the Disrupt CAD III, a prospective, single-arm multicenter trial of coronary IVL, periprocedural MI rate varied depending on the event definition. It was 7.3% using the Fourth Universal Definition, 6.8% using a sensitive definition (peak post-PCI CK-MB levels > 3 × URL), and significantly lower (2.6%) with the SCAI definition (3). Compared with the Disrupt CAD III, the rate of periprocedural MI in our study was similar (6.7 vs. 7.3%) when the Fourth Universal Definition was used and higher when assessed with the sensitive (20 vs. 6.8%) and SCAI definition (6.7 vs. 2.6%). No significant difference was found between groups, regardless of MI definition (Table 6). The higher event rate when two of the periprocedural MI definitions were used may be justified by the higher complexity of the coronary lesions treated in our real-world patients. Indeed, a sizable number of them fulfilled Disrupt CAD III exclusion criteria (multivessel PCI, ISR, extremely tortuous target vessels, ostial location, left main disease, bifurcation lesions, lesions > 40 mm, tandem lesions, and acute coronary syndrome) (3).

### Late Major Adverse Cardiovascular Event

At long-term (median 12.9 months, IQR 8.6–20.1 months) follow-up, we observed only 1 (0.95%) sudden cardiac death 8 days after a TLR for ISR, probably because of ST. Overall, the long-term MACE rate was 13.3%, driven by TVR occurring in 13 (11.8%) lesions, with 5 (4.8%) TVMI and 1 (0.9%) definite ST. Overall, 8 (7.3%) lesions underwent TLR because of ISR at a median follow-up of 10.1 months (IQR 5–13.5). These results are acceptable if compared with previous trials that used rotational atherectomy for treating similar lesions and reporting a TLR rate ranging from 8.2 to 16.6% (20–26). ISR is related to multiple clinical and angiographical factors (vessel size, lesion length, lesion calcification, postprocedural residual stenosis, and length of stented vessel) (27). Subgroup analysis (Supplementary Tables 7–12) showed that none of these factors were more prevalent in the TLR group of our cohort while belonging to the ISR–IVL group was the strongest predictor (Table 7; *p* = 0.006). This suggests that, despite the acute angiographic success, the long-term outcomes might be less favorable when IVL is used in patients with stent under expansion. The high MACE rates in this subgroup highlight the challenges of this subset of patients and remarks that even IVL plus DCB cannot be advocated as definitive therapy. This is relevant since IVL use in ISR is off-label, even if it is commonly employed in this scenario.

Another interesting finding of our analysis is that the avoidance of high-pressure predilatation before IVL was identified as the only additional independent predictor of TLR (Table 7; *p* = 0.04). According to this, a non-significant higher TLR rate was observed in P-IVL (Table 6). This finding is difficult to explain. Indeed, high-pressure dilatation before IVL could elicit stronger vascular damage and inflammatory response, and also an increased risk of rupture of the internal elastic lamina, thus, promoting a stronger vessel reaction (28). Further studies should address this issue to verify if a possible correlation exists between a primary or a secondary IVL approach and subsequent risk of stent failure.

### Periprocedural Complications

The secondary safety endpoint (freedom from IVL-related procedural complications) was met in 97 (92.4%) patients with 102 (92.7%) lesions. Serious angiographic complications were rare, consisting in 3 (2.7%) flow-limiting dissections resolved by DES implantation and 5 (4.5%) ruptures of the IVL balloon that in 1 (0.9%) case caused Type III vessel perforation successfully managed by covered stent implantation. These data are in line with those of prior studies (3–6, 14) confirming that coronary IVL is associated with a low rate of procedural complications with infrequent clinical sequelae. Nevertheless, since complications may occur, we think that IVL should not be used in unmonitored settings.

### Limitations

This study has several limitations.

First, this is a single-center retrospective, observational registry of patients with moderately or severely calcified coronary lesions and may not be representative of the general population treated by IVL.

Second, IVL was used for different indications, some of which (B-IVL and ISR-IVL groups) are still off-label. Depending on the design of the study, a standard approach was not required for the treatment of *de novo* lesions; therefore, the PCI strategy adopted in each case may differ from that used in other centers. The small number of patients assigned to the various groups may have affected the results. Thus, larger studies are needed to better assess safety, effectiveness, and long-term outcome in patients undergoing IVL with different indications.

Third, in this “real-world” experience we found that only a minority of procedures were guided by IVUS or OCT. Intravascular imaging plays a key role in guiding complex procedures, improving both acute and long-term outcomes, and providing insights into the mechanism of IVL in facilitating stent expansion. This is especially true when IVL is used for off-label indications, such as ISR. In this report, only 47.8% of lesions in the ISR–IVL underwent imaging-guided procedures, and this may partly explain the higher rate of stent failure observed in this group. Larger studies with intravascular imaging are warranted to provide insights into the mechanism of IVL in facilitating stent expansion in the setting of different IVL indications.

Third, the median follow-up was only 12.9 months. Because late events may be more frequent after treatment of complex calcified coronary lesions, longer-term follow-up will be needed, and is currently ongoing at our center.

## Conclusion

Our data suggest that IVL is a feasible and safe technique for treating moderately or severely calcified coronary lesions. Similar acute results are observed in *de novo* and ISR lesions, regardless of the clinical presentation and IVL indication. Periprocedural MI and IVL-related complications might occur, but are rare and not associated with relevant MACE. An acceptable rate of late events may occur, but they are more frequent when IVL is performed for ISR related to calcium-mediated stent under expansion.

## Data Availability Statement

The datasets presented in this article are not readily available because due to the nature of this research, participants of this study did not agree for their data to be shared publicly. Requests to access the datasets should be directed to Angelo Mastrangelo, mastrangelongl@gmail.com.

## Ethics Statement

Ethical review and approval was not required for the study on human participants in accordance with the Local Legislation and Institutional Requirements. The patients/participants provided their written informed consent to participate in this study.

## Author Contributions

AM, GM, SG, and AB contributed to the conception and design of the study. AM and SG organized the database. AM and MC performed the statistical analysis. AM wrote the first draft of the manuscript. GM, SG, and AB wrote sections of the manuscript. All authors contributed to manuscript revision, read, and approved the submitted version of the manuscript.

## Conflict of Interest

The authors declare that the research was conducted in the absence of any commercial or financial relationships that could be construed as a potential conflict of interest.

## Publisher's Note

All claims expressed in this article are solely those of the authors and do not necessarily represent those of their affiliated organizations, or those of the publisher, the editors and the reviewers. Any product that may be evaluated in this article, or claim that may be made by its manufacturer, is not guaranteed or endorsed by the publisher.
