# Supplementary material for: Intravascular Lithotripsy in Calcified Coronary Lesions: A Single-Center Experience in “Real-World” Patients
Source: Front Cardiovasc Med. 2022 Feb 21;9:829117. doi: 10.3389/fcvm.2022.829117 (PMC8900981; doi:10.3389/fcvm.2022.829117)
Supplement: Supplementary file 1 [file Table_1.DOCX]

***Supplementary Material***

**Supplementary Tables**

**Supplementary Table 1. Baseline characteristics of patients who met or did not meet primary effectiveness endpoint**

|  | **Primary endpoint met** | **Primary endpoint not met** | ***p*** |
| --- | --- | --- | --- |
| **No. of patients, n** | 88 | 17 | – |
| Male, n (%) | 73 (82.95) | 16 (94.12) | 0.24 |
| Age (mean±SD), n | 71.6±7.5 | 70±8.1 | 0.42 |
| **Risk Factors** |  |  |  |
| Obesity, * n (%) | 13 (14.8) | 3 (17.6) | 0.76 |
| Hypertension, n (%) | 65 (73.9) | 13 (76.5) | 0.82 |
| Hypercholesterolemia, n (%) | 64 (72.7) | 15 (88.2) | 0.18 |
| Smoking, n (%) | 30 (34.1) | 9 (52.9) | 0.14 |
| Family history of CVD, n (%) | 22 (25) | 8 (47.1) | 0.07 |
| Diabetes mellitus, n (%) | 27 (30.7) | 2 (11.8) | 0.11 |
| History of CAD, n (%) | 56 (63.6) | 9 (52.9) | 0.41 |
| Prior MI, n (%) | 29 (33) | 4 (23.5) | 0.44 |
| Prior CABG, n (%) | 16 (18.2) | 1 (5.9) | 0.21 |
| Prior PCI, n (%) | 45 (51.1) | 7 (41.2) | 0.45 |
| LVEF (mean±SD), % | 57±9 | 59±5 | 0.43 |
| Prior stroke, n (%) | 3 (3.4) | 2 (11.8) | 0.13 |
| Chronic kidney disease, † n (%) | 19 (21.6) | 7 (41.2) | 0.09 |
| Creatinine (mean±SD), mg/dl | 1.05±0.38 | 1.22±0.60 | 0.13 |
| End-stage renal disease, n (%) | 1 (1.1) | 0 (0) | 0.66 |
| Peripheral artery disease, n (%) | 24 (27.3) | 4 (23.5) | 0.75 |
| **Clinical Presentation** |  |  |  |
| Chronic coronary syndrome, n (%) | 77 (87.5) | 16 (94.1) | 0.43 |
| Acute coronary syndrome, n (%) | 11 (12.5) | 1 (5.9) | 0.43 |

Values are mean±SD or n (%).

CABG: coronary artery bypass grafting; CAD: coronary artery disease; CT: computer tomography; CVD: cardiovascular disease; ISR: in-stent restenosis; IVL: intravascular lithotripsy; LVEF: left ventricular ejection fraction; MI: myocardial infarction; PCI: percutaneous coronary intervention; TAVR/AVR: transcatheter aortic valve replacement/aortic valve replacement.

* Defined as body max index >30 kg/m^2^. † Defined as glomerular filtration rate <60 mL/min.

**Supplementary Table 2. Lesion characteristics of patients who met or did not meet primary effectiveness endpoint**

|  | **Primary endpoint met** | **Primary endpoint not met** | ***p*** |
| --- | --- | --- | --- |
| **Number of IVL Treated Lesions, n (%)** | 93 (100) | 17 (100) | – |
| **CAD type** | | | |
| One-vessel CAD, n (%) | 22 (23.7) | 4 (23.5) | 0.99 |
| Two-vessel CAD, n (%) | 36 (38.7) | 7 (41.2) | 0.85 |
| Three-vessel CAD, n (%) | 35 (37.6) | 6 (35.3) | 0.85 |
| LM disease, * n (%) | 18 (19.4) | 4 (23.5) | 0.69 |
| **No. of Treated Vessels during index-procedure** | | | |
| One-vessel PCI, n (%) | 52 (55.9) | 7 (41.2) | 0.26 |
| Two-vessels PCI, n (%) | 35 (37.6) | 9 (52.9) | 0.24 |
| Three-vessels PCI, n (%) | 6 (6.5) | 1 (5.9) | 0.93 |
| **IVL – Treated Vessel** | | | |
| LM, n (%) | 10 (10.8) | 1 (5.9) | 0.54 |
| LAD, n (%) | 49 (52.7) | 7 (41.2) | 0.38 |
| CFX, n (%) | 12 (12.9) | 2 (11.8) | 0.90 |
| RCA, n (%) | 22 (23.7) | 7 (41.2) | 0.13 |
| **Lesion Localization** | | | |
| Ostial, n (%) | 15 (16.1) | 1 (5.9) | 0.27 |
| Proximal, n (%) | 44 (47.3) | 10 (58.8) | 0.38 |
| Medial, n (%) | 23 (24.7) | 5 (29.4) | 0.68 |
| Distal, n (%) | 11 (11.8) | 1 (5.9) | 0.47 |
| **Lesion Characteristics** | | | |
| Ellis A, † n (%) | 0 (0) | 0 (0) | – |
| Ellis B1, † n (%) | 1 (1.1) | 1 (5.9) | 0.17 |
| Ellis B2, † n (%) | 32 (34.4) | 6 (35.5) | 0.94 |
| Ellis C, † n (%) | 40 (43) | 6 (35.3) | 0.55 |
| ISR, n (%) | 19 (20.4) | 4 (23.5) | 0.77 |
| In-stent thrombosis, n (%) | 1 (1.1) | 0 (0) | 0.67 |
| Bifurcation lesions, n (%) | 20 (21.5) | 1 (5.9)) | 0.13 |
| CTO, n (%) | 1 (1.1) | 1 (5.9) | 0.17 |
| **Coronary Calcification** | | | |
| Moderate, n (%) | 12 (12.9) | 4 (23.5) | 0.25 |
| Severe, n (%) | 81 (87.1) | 13 (76.5) | 0.25 |
| **Lesion Assessment** | | | |
| Eccentric, n (%) | 70 (75.3) | 12 (70.6) | 0.684 |
| Concentric, n (%) | 23 (24.7) | 5 (29.4) | 0.684 |
| **Lesion Access** | | | |
| Proximal tortuosity, n (%) | 16 (17.2) | 3 (17.6) | 0.97 |
| **Procedural Characteristics** | | | |
| Radial artery, n (%) | 72 (77.4) | 14 (82.4) | 0.65 |
| Femoral artery, n (%) | 26 (28) | 4 (23.5) | 0.71 |
| 7F catheters, n (%) | 6 (6.5) | 2 (11.8) | 0.44 |
| Guide-Catheter Extension, n (%) | 25 (26.9) | 5 (29.4) | 0.83 |
| Protected PCI, n (%) | 4 (4.3) | 0 (0) | 0.38 |
| IVUS guided PCI, n (%) | 21 (22.6) | 5 (29.4) | 0.54 |
| OCT guided PCI, n (%) | 10 (10.8) | 2 (11.8) | 0.90 |
| Contrast agent (mean±SD), mL | 270±134 | 240±107 | 0.39 |
| Fluoroscopy time (mean±SD), min | 22.82±10.14 | 30.97±26.12 | 0.03 |
| Total DAP (mean±SD), CGy$\times$cm^2^ | 13,662±8,113 | 17,176±12,490 | 0.13 |

Values are mean ± SD or n (%)

CAD: coronary artery disease; CFX: circumflex; CTO: chronic total occlusion; DAP: dose-area product, FFR: fractional flow reserve; ISR: in-stent restenosis; IVL: intravascular lithotripsy; IVUS: intravascular ultrasound; LAD: left anterior descending; LM: left main; OCT: optical coherence tomography; PCI: percutaneous coronary intervention; RCA: right coronary artery.

* Defined as diameter stenosis >50%. † American College of Cardiology-American Heart Association classification.

**Supplementary Table 3. Procedural characteristics of patients who met or did not meet primary effectiveness endpoint**

|  | **Primary endpoint met** | **Primary endpoint not met** | ***p*** |
| --- | --- | --- | --- |
|  | **n = 93** | **n = 17** |  |
| **IVL** | | | |
| Number of pulses (mean±SD), n | 64±20 | 61±25 | 0.77 |
| Diameter of IVL balloon (mean±SD), mm | 3.0±0.5 | 2.9±0.4 | 0.49 |
| **Predilatation** | | | |
| Pre-IVL high-pressure dilation, n (%) | 58 (62.4) | 12 (70.6) | 0.52 |
| Largest diameter (mean±SD), mm | 2.8±0.4 | 3.0±0.6 | 0.32 |
| Mean pressure (mean±SD), atm | 19±4 | 18±5 | 0.56 |
| Pre-IVL cutting balloon, n (%) | 6 (6.5) | 1 (5.9) | 0.93 |
| Largest balloon diameter (mean±SD), mm | 2.8±0.4 | 3.25 | – |
| Post-IVL high-pressure dilation, n (%) | 55 (59.1) | 10 (58.8) | 0.93 |
| Largest balloon diameter (mean±SD), mm | 3.1±0.4 | 3.3±0.6 | 0.13 |
| Mean pressure (mean±SD), atm | 20±8 | 20±8.6 | 0.81 |
| No. of balloons/lesion (mean±SD), n | 3.1±2.1 | 2.5±1.9 | 0.26 |
| **Lesion treatment** | | | |
| DES, n (%) | 79 (84.9) | 12 (70.6) | 0.15 |
| Total stented length (mean±SD), mm | 35.7±20.7 | 34.4±20 | 0.84 |
| DCB, n (%) | 11 (11.8) | 1 (5.9) | 0.47 |
| **Postdilatation** | | | |
| High-pressure dilation before IVL, n (%) | 8 (8.6) | 3 (17.6) | 0.25 |
| Largest balloon diameter (mean±SD), mm | 3.1±0.4 | 3.3±0.3 | 0.33 |
| Mean pressure (mean±SD), atm | 22±9 | 23±1 | 0.80 |
| Final high-pressure dilation, n (%) | 72 (77.4) | 12 (70.6) | 0.54 |
| Largest balloon diameter (mean±SD), mm | 3.6±0.6 | 3.5±0.6 | 0.48 |
| Mean pressure (mean±SD), atm | 19±5 | 19±3 | 0.89 |

Values are mean ± SD or n (%).

DCB: drug-coated balloon; DES: drug-eluting stent; ISR: in-stent restenosis; IVL: intravascular lithotripsy.

**Supplementary Table 4. Quantitative coronary angiography results of patients who met or did not meet primary effectiveness endpoint**

|  | **Primary endpoint met** | **Primary endpoint not met** | ***p*** |
| --- | --- | --- | --- |
|  | **n = 93** | **n = 17** |  |
| **Reference Vessel** | | | |
| Diameter (mean±SD), mm | 3.25±0.63 | 3.32±0.66 | 0.72 |
| No. of lesions with RVD >3 mm, n (%) | 58 (62.4) | 12 (70.6) | 0.52 |
| **Lesion – Baseline Assessment** | | | |
| Length (mean±SD), mm | 19.96±15.72 | 23.29±17.61 | 0.43 |
| No. of long lesions, * n (%) | 46 (49.5) | 10 (58.8) | 0.48 |
| MLD (mean±SD), mm | 0.98±0.47 | 0.97±0.42 | 0.94 |
| DS (mean±SD), % | 70.04±12.66 | 70.41±11.11 | 0.91 |
| **Lesion – Final Assessment** | | | |
| MLD (mean±SD), mm | 2.88±0.56 | 2.21±0.73 | <.0001 |
| DS (mean±SD), % | 11.59±5.66 | 33.24±15.83 | <.0001 |
| Acute Gain, mm | 1.89±0.55 | 1.24±0.59 | <.0001 |

Values are mean ± SD or n (%).

DS: diameter stenosis; ISR: in-stent restenosis; IVL: intravascular lithotripsy; MLD: minimal lumen diameter.

* Defined as lesion length >15 mm.

**Supplementary Table 5. Effectiveness Endpoints of of patients who met or did not meet primary effectiveness endpoint**

|  | **Primary endpoint met** | **Primary endpoint not met** | ***p*** |
| --- | --- | --- | --- |
|  | **n = 93** | **n = 17** |  |
| IVL device delivery and IVL treatment, n (%) | 93 (100) | 16 (94.1) | 0.15 |
| Stent successful expansion, n (%) | 93 (100) | 14 (82.4) | 0.003 |
| Final TIMI 3 flow, n (%) | 93 (100) | 17 (100) | – |
| Residual DS <50%, n (%) | 93 (100) | 14 (82.4) | 0.003 |

Values are n (%).

DS: diameter stenosis; ISR: in-stent restenosis; IVL: intravascular lithotripsy; TIMI: Thrombolysis in Myocardial Infarction.

**Supplementary Table 6. Safety endpoints of patients of patients who met or did not meet primary effectiveness endpoint**

|  | **Primary endpoint met**  **n = 88** | **Primary endpoint not met**  **n = 17** | | ***p*** |
| --- | --- | --- | --- | --- |
| **In-hospital MACE** | | | | |
| Cardiac death, n (%) | 0 (0) | | 0 (0) | – |
| Periprocedural MI (CK-MB >3$\times$URL), n (%) | 19 (21.6) | | 2 (11.8) | 0.35 |
| Periprocedural MI (IV UD definition), n (%) | 7 (7.9) | | 0 (0) | 0.59 |
| Periprocedural MI (SCAI definition), n (%) | 7 (7.9) | | 0 (0) | 0.59 |
| Stent thrombosis,* n (%) | 0 (0) | | 0 (0) | – |
| Target lesion revascularization,* n (%) | 0 (0) | | 0 (0) | – |
| Target vessel revascularization,* n (%) | 0 (0) | | 0 (0) | – |
| **Early MACE (<30 days)** | 0 (0) | | 0 (0) | – |
| Cardiac death, n (%) | 0 (0) | | 0 (0) | – |
| Spontaneous MI, n (%) | 0 (0) | | 0 (0) | – |
| Stent thrombosis,* n (%) | 0 (0) | | 0 (0) | – |
| Target lesion revascularization,* n (%) | 0 (0) | | 0 (0) | – |
| Target vessel revascularization,* n (%) | 0 (0) | | 0 (0) | – |
| **Late MACE (>30 days)** | 14 (15.9) | | 0 (0) | 0.12 |
| Cardiac death, n (%) | 2 (2.3) | | 0 (0) | 1 |
| Spontaneous MI (TVMI), n (%) | 5 (5.7) | | 0 (0) | 0.59 |
| Stent thrombosis,* n (%) | 2 (2.3) | | 0 (0) | 1 |
| Probable, n (%) | 1 (1.1) | | 0 (0) | 1 |
| Definite, n (%) | 1 (1.1) | | 0 (0) | 1 |
| Target vessel revascularization,* n (%) | 13 (13.9) | | 0 (0) | 0.21 |
| Target lesion revascularization,* n (%) | 8 (8.6) | | 0 (0) | 0.35 |
| Target vessel non-target lesion revascularization, * n (%) | 7 (7.5) | | 0 (0) | 0.59 |
| **Secondary Safety Endpoint** | | | | |
| Freedom from relevant procedural complications, n (%) | 81 (92.1) | | 16 (94.1) | 0.77 |
| Serious angiographic complications,* n (%) | 3 (3.2) | | 1 (5.9) | 0.49 |
| Flow-limiting dissection,* n (%) | 2 (2.2) | | 1 (5.9) | 0.36 |
| Perforation,* n (%) | 1 (1.1) | | 0 (0) | 1 |
| Acute stent thrombosis,* n (%) | 0 (0) | | 0 (0) | – |
| Persistent slow-flow or no-reflow phenomena,* n (%) | 0 (0) | | 0 (0) | – |
| Serious IVL-related arrhythmias,* n (%) | 0 (0) | | 0 (0) | – |
| Ventricular tachycardia,* n (%) | 0 (0) | | 0 (0) | – |
| Ventricular fibrillation,* n (%) | 0 (0) | | 0 (0) | – |
| Failure of the IVL system,* n (%) | 5 (5.4) | | 0 (0) | 1 |
| Malfunction,* n (%) | 0 (0) | | 0 (0) | – |
| IVL balloon burst,* n (%) | 5 (5.4) | | 0 (0) | 1 |

Values are n (%).

ISR: in-stent restenosis; IVL: intravascular lithotripsy; MI: myocardial infarction; SCAI: Society for Cardiovascular Angiography and Intervention; TVMI: target vessel myocardial infarction

* Calculations were based on a per lesion basis (Primary endpoint met n=93, Primary endpoint not met n=17)

**Supplementary Table 7. Baseline Characteristics in Patients with and without TLR**

|  | **TLR** | **No TLR** | ***p*** |
| --- | --- | --- | --- |
| No. of patients (%) | 8 (100) | 97 (100) | – |
| Male, n (%) | 6 (75) | 83 (85.6) | 0.42 |
| Age (mean±SD), n | 71.4±8.8 | 71.4±7.5 | 0.99 |
| Risk Factors |  |  |  |
| Obesity, * n (%) | 2 (25) | 14 (14.4) | 0.42 |
| Hypertension, n (%) | 8 (100) | 70 (72.2) | 0.08 |
| Hypercholesterolemia, n (%) | 7 (87.5) | 72 (74.2) | 0.41 |
| Smoking, n (%) | 4 (50) | 48 (49.5) | 0.98 |
| Family history of CVD, n (%) | 1 (12.5) | 29 (29.9) | 0.30 |
| Diabetes mellitus, n (%) | 2 (25) | 27 (27.8) | 0.86 |
| History of CAD, n (%) | 8 (100) | 57 (58.8) | 0.02 |
| Prior MI, n (%) | 5 (62.5) | 28 (28.9) | 0.049 |
| Prior CABG, n (%) | 0 (0) | 17 (17.5) | 0.35 |
| Prior PCI, n (%) | 7 (87.5) | 45 (46.4) | 0.025 |
| LVEF (mean±SD), % | 59±3 | 58±9 | 0.66 |
| Prior stroke, n (%) | 0 (0) | 5 (5.2) | 1 |
| Chronic kidney disease, † n (%) | 3 (37.5) | 23 (23.7) | 0.39 |
| Creatinine (mean±SD), mg/dl | 1.03±0.20 | 1.08±0.44 | 0.77 |
| End-stage renal disease, n (%) | 0 (0) | 1 (1) | 1 |
| Peripheral artery disease, n (%) | 2 (25) | 26 (26.8) | 0.91 |
| Clinical Presentation |  |  |  |
| Chronic coronary syndrome, n (%) | 4 (50) | 89 (91.8) | <.0001 |
| Acute coronary syndrome, n (%) | 4 (50) | 8 (8.2) | <.0001 |

Values are mean±SD or n (%).

CABG:coronary artery bypass grafting; CAD: coronary artery disease; CVD: cardiovascular disease; ISR: in-stent restenosis; IVL: intravascular lithotripsy; LVEF: left ventricular ejection fraction; MI: myocardial infarction; PCI: percutaneous coronary intervention; TAVR/AVR: transcatheter aortic valve replacement/aortic valve replacement.

* Defined as body max index >30 kg/m^2^. † Defined as glomerular filtration rate <60 mL/min.

**Supplementary Table 8. Lesion Characteristics in Patients with and without TLR**

|  | **TLR** | **No TLR** | ***p*** |
| --- | --- | --- | --- |
| Number of Treated Lesions (%) | 8 (100) | 102 (100) | – |
| **CAD type** | | | |
| One-vessel PCI, n (%) | 2 (25) | 24 (23.5) | 0.93 |
| Two-vessel PCI, n (%) | 4 (50) | 39 (38.2) | 0.51 |
| Three-vessel PCI, n (%) | 2 (25) | 39 (38.2) | 0.46 |
| LM disease, n (%) | 1 (12.5) | 21 (20.6) | 0.58 |
| **No. of treated vessels during index-procedure** | | | |
| One-vessel PCI, n (%) | 5 (62.5) | 54 (52.9) | 0.60 |
| Two-vessels PCI, n (%) | 2 (25) | 42 (41.2) | 0.37 |
| Three-vessels PCI, n (%) | 1 (12.5) | 6 (5.9) | 0.46 |
| **IVL – treated vessel** | | | |
| LM, n (%) | 1 (12.5) | 10 (9.8) | 0.81 |
| LAD, n (%) | 3 (37.5) | 53 (52) | 0.43 |
| CFX, n (%) | 0 (0) | 14 (13.7) | 0.59 |
| RCA, n (%) | 4 (50) | 25 (24.5) | 0.12 |
| **Lesion Location** | | | |
| Ostial, n (%) | 1 (12.5) | 15 (14.7) | 0.87 |
| Proximal, n (%) | 3 (37.5) | 51 (50) | 0.50 |
| Medial, n (%) | 3 (37.5) | 25 (24.5) | 0.417 |
| Distal, n (%) | 1 (12.5) | 11 (10.8) | 0.88 |
| **Lesion Characteristics** | | | |
| Ellis A, † n (%) | 0 (0) | 0 (0) | – |
| Ellis B1, † n (%) | 0 (0) | 2 (2) | 1 |
| Ellis B2, † n (%) | 1 (12.5) | 37 (36.3) | 0.17 |
| Ellis C, † n (%) | 2 (25) | 44 (43.1) | 0.32 |
| ISR, n (%) | 5 (62.5) | 18 (17.6) | 0.002 |
| Stent thrombosis, n (%) | 0 (0) | 1 (1) | 1 |
| Bifurcation lesions, n (%) | 1 (12.5) | 20 (19.6) | 0.62 |
| CTO, n (%) | 0 (0) | 2 (2) | 1 |
| **Coronary Calcification** | | | |
| Moderate, n (%) | 1 (12.5) | 15 (14.7) | 0.87 |
| Severe, n (%) | 7 (87.5) | 87 (85.3) | 0.87 |
| **Lesion Characteristics** | | | |
| Eccentric, n (%) | 6 (75) | 76 (74.5) | 0.98 |
| Concentric, n (%) | 2 (25) | 26 (25.5) | 0.98 |
| **Lesion Access** | | | |
| Severe tortuosity, n (%) | 1 (12.5) | 18 (17.6) | 0.71 |
| **Procedural Characteristics** | | | |
| Radial artery, n (%) | 8 (100) | 78 (76.5) | 0.12 |
| Femoral artery, n (%) | 0 (0) | 30 (29.4) | 0.1 |
| 7F catheter, n (%) | 0 (0) | 8 (7.8) | 1 |
| Guide-catheter extension, n (%) | 2 (25) | 28 (27.5) | 0.88 |
| Protected PCI, n (%) | 0 (0) | 4 (3.9) | 1 |
| IVUS-guided PCI, n (%) | 2 (25) | 24 (23.5) | 0.93 |
| OCT-guided PCI, n (%) | 1 (12.5) | 11 (10.8) | 0.88 |
| Contrast agent (mean±SD), mL | 252±203 | 266±124 | 0.77 |
| Fluoroscopy time (mean±SD), min | 21,25±10.74 | 24.3±13.2 | 0.55 |
| Total DAP (mean±SD), CGy$\times$cm^2^ | 10,055±5,356 | 14,530±9,108 | 0.17 |

Values are mean±SD or n (%).

CAD: coronary artery disease; CFX: circumflex; CTO: chronic total occlusion; DAP: dose-area product; FFR: fractional flow reserve; ISR: in-stent restenosis; IVL: intravascular lithotripsy; IVUS: intravascular ultrasound; LAD: left anterior descending; LM: left main; OCT: optical coherence tomography; PCI: percutaneous coronary intervention; RCA: right coronary artery.

* Defined as diameter stenosis >50%. † American College of Cardiology - American Heart Association classification.

**Supplementary Table 9. Procedural characteristics in patients with and without TLR**

|  | **TLR** | **No TLR** | ***p*** |
| --- | --- | --- | --- |
|  | **n = 8** | **n = 102** |  |
| **IVL** | | | |
| Number of pulses (mean±SD), n | 74±18 | 63±21 | 0.15 |
| Diameter of IVL balloon (mean±SD), mm | 3.1±0.5 | 3.0±0.4 | 0.59 |
| **Predilatation** | | | |
| Pre-IVL high-pressure dilation, n (%) | 2 (25) | 68 (66.7) | 0.02 |
| Largest balloon diameter (mean±SD), mm | 2.5 ± 0 | 2.9±0.4 | 0.21 |
| Mean pressure (mean±SD), atm | 17 ± 1 | 19±4 | 0.55 |
| Pre-IVL cutting balloon, n (%) | 1 (12.5) | 6 (5.9) | 0.46 |
| Largest balloon diameter (mean±SD), mm | 2.5 | 3.0 ± 0.4 | - |
| Post-IVL high-pressure dilation, n (%) | 7 (87.5) | 58 (56.9) | 0.21 |
| Largest balloon diameter (mean±SD), mm | 3.3±0.7 | 3.1±0.4 | 0.32 |
| Mean pressure (mean±SD), atm | 25±13 | 20±7 | 0.09 |
| No. of balloons/lesion (mean±SD), n | 3.6±2.1 | 3.0±2.0 | 0.41 |
| **Lesion treatment** | | | |
| DES, n (%) | 5 (62.5) | 86 (84.3) | 0.12 |
| Total stented length (mean±SD), mm | 27.8±17.74 | 35.95±20.66 | 0.39 |
| DCB, n (%) | 3 (37.5) | 9 (8.8) | 0.01 |
| **Postdilatation** | | | |
| High-pressure dilation before IVL, n (%) | 0 (0) | 11 (10.8) | 1 |
| Largest balloon diameter (mean±SD), mm | – | 3.1±0.4 | – |
| Mean pressure (mean±SD), atm | – | 22±7 | – |
| Final high-pressure dilation, n (%) | 5 (62.5) | 79 (77.5) | 0.34 |
| Largest balloon diameter (mean±SD), mm | 3.7±0.4 | 3.6±0.6 | 0.76 |
| Mean pressure (mean±SD), atm | 19±1 | 19±5 | 0.99 |

Values are mean±SD or n (%).

DCB: drug-coated balloon; DES: drug-eluting stent; ISR: in-stent restenosis; IVL: intravascular lithotripsy.

**Supplementary Table 10. Quantitative coronary angiography results in patients with and without TLR**

|  | **TLR** | **No TLR** | ***p*** |
| --- | --- | --- | --- |
|  | **n = 8** | **n = 102** |  |
| **Reference Vessel** | | | |
| Diameter (mean±SD), mm | 3.30 ± 0.67 | 3.26 ± 0.64 | 0.85 |
| No. of lesions with RVD >3 mm, n (%) | 5 (62.5) | 65 (63.7) | 0.95 |
| **Lesion – Basal Assessment** | | | |
| Length (mean±SD), mm | 20.38 ± 12.4 | 20.47 ± 16.28 | 0.99 |
| No. of long lesions,* n (%) | 4 (50) | 52 (51) | 0.96 |
| MLD (mean±SD), mm | 0.88 ± 0.63 | 0.99 ± 0.45 | 0.51 |
| DS (mean±SD), % | 75.25 ± 16.42 | 69.7 ± 12.02 | 0.22 |
| **Lesion – Final Assessment** | | | |
| MLD (mean±SD), mm | 2.95 ± 0.52 | 2.76 ± 0.63 | 0.42 |
| DS (mean±SD), % | 10.13 ± 5.84 | 15.31 ± 11.45 | 0.21 |
| Acute Gain, mm | 2.08 ± 0.44 | 1.8 ± 0.6 | 0.17 |

Values are mean±SD or n (%).

DS: diameter stenosis; ISR: in-stent restenosis; IVL: intravascular lithotripsy; MLD: minimal lumen diameter.

* Defined as lesion length >15 mm.

**Supplementary Table 11. Effectiveness Endpoints in Patients with and without TLR**

|  | **TLR** | **No TLR** | ***p*** |
| --- | --- | --- | --- |
|  | **n = 8** | **n = 102** |  |
| IVL device delivery and IVL treatment, n (%) | 8 (100) | 101 (99) | 0.78 |
| Stent successful expansion, n (%) | 8 (100) | 99 (97.1) | 0.62 |
| Final TIMI 3 flow, n (%) | 8 (100) | 102 (100) | – |
| Residual DS <20%, n (%) | 8 (100) | 85 (83.3) | 0.21 |
| Residual DS <50%, n (%) | 8 (100) | 99 (97.1) | 0.62 |

Values are n (%).

DS: diameter stenosis; ISR: in-stent restenosis; IVL: intravascular lithotripsy; TIMI: Thrombolysis in Myocardial Infarction.

**Supplementary Table 12. Safety endpoints in patients with and without TLR**

|  | **TLR** | **No TLR** | ***p*** |
| --- | --- | --- | --- |
|  | **n = 8** | **n = 97** |  |
| **In-hospital MACE** | | | |
| Cardiac death, n (%) | 0 (0) | 0 (0) | – |
| Periprocedural MI (CK-MB >3$\times$URL), n (%) | 3 (37.5) | 18 (18.6) | 0.19 |
| Periprocedural MI (IV UD), n (%) | 2 (25) | 5 (5.2) | 0.03 |
| Periprocedural MI (SCAI definition), n (%) | 2 (25) | 5 (5.2) | 0.03 |
| Stent thrombosis,* n (%) | 0 (0) | 0 (0) | – |
| Target lesion revascularization,* n (%) | 0 (0) | 0 (0) | – |
| Target vessel revascularization,* n (%) | 0 (0) | 0 (0) | – |
| **Early MACE (<30 days)** | 0 (0) | 0 (0) | – |
| Cardiac death, n (%) | 0 (0) | 0 (0) | – |
| Spontaneous MI | 0 (0) | 0 (0) | – |
| Stent thrombosis,* n (%) | 0 (0) | 0 (0) | – |
| Target lesion revascularization,* n (%) | 0 (0) | 0 (0) | – |
| Target vessel revascularization,* n (%) | 0 (0) | 0 (0) | – |
| **Late MACE (>30 days)** |  |  |  |
| Cardiac death, n (%) | 1 (12.5) | 1 (1) | 0.02 |
| Spontaneous MI (TVMI), n (%) | 3 (37.5) | 2 (2.1) | <.0001 |
| Stent thrombosis,* n (%) | 1 (12.5) | 1 (1) | 0.02 |
| Probable,* n (%) | 1 (12.5) | 0 (0) | 0.07 |
| Definite,* n (%) | 0 (0) | 1 (1) | 0.07 |
| Target vessel revascularization,* n (%) | 8 (100) | 5 (4.9) | <.0001 |
| Target vessel nontarget site revascularization,* n (%) | 2 (25) | 5 (4.9) | 0.02 |
| **Secondary Safety Endpoint** | | | |
| Freedom from relevant procedural complications, n (%) | 6 (75) | 91 (93.8) | 0.05 |
| Serious angiographic complications,* n (%) | 2 (25) | 6 (5.9) | 0.04 |
| Flow-limiting dissection,* n (%) | 1 (12.5) | 2 (2) | 0.07 |
| Perforation,* n (%) | 0 (0) | 1 (1) | 0.07 |
| Acute stent thrombosis,* n (%) | 0 (0) | 0 (0) | – |
| Persistent slow-flow or no-reflow phenomena,* n (%) | 0 (0) | 0 (0) | – |
| Serious IVL-related arrhythmias,* n (%) | 0 (0) | 0 (0) | – |
| Ventricular tachycardia,* n (%) | 0 (0) | 0 (0) | – |
| Ventricular fibrillation,* n (%) | 0 (0) | 0 (0) | – |
| Failure of the IVL system,* n (%) | 1 (12.5) | 4 (3.9) | 0.28 |
| Malfunction,* n (%) | 0 (0) | 0 (0) | – |
| IVL balloon burst balloon,* n (%) | 1 (12.5) | 4 (3.9) | 0.28 |

Values are n (%).

ISR: in-stent restenosis; IVL: intravascular lithotripsy; MI: myocardial infarction; SCAI: Society for Cardiovascular Angiography and Intervention; TVMI: target vessel myocardial infarction.

*Calculations were based on a per lesion basis (TLR=8, no TLR=102).

**Supplementary Table 13. Antithrombotic therapy in the overall population and patient subgroups**

|  | **Overall** | **Primary IVL** | **Secondary IVL** | **Bailout IVL** | **ISR IVL** |
| --- | --- | --- | --- | --- | --- |
|  |  |  |  |  |  |
| **No. of patients** | 105 | 24 | 48 | 11 | 22 |
| **Antiplatelets only** | 98 (93.3) | 22 (91.7) | 45 (93.8) | 11 (100) | 20 (90.9) |
| Aspirin + Clopidogrel | 86 (81.9) | 21 (87.5) | 42 (87.5) | 9 (81.8) | 14 (63.6) |
| Aspriri + Ticagrelor | 5 (4.8) | 0 (0) | 2 (4.2) | 0 (0) | 3 (13.6) |
| Aspirin + Prasugrel | 2 (1.9) | 1 (4.2) | 0 (0) | 0 (0) | 1 (4.5) |
| Indobufen + Clopidogrel | 3 (2.9) | 0 (0) | 1 (2.1) | 2 (18.2) | 0 (0) |
| Indobufen + Ticagrelor | 1 (0.95) | 0 (0) | 0 (0) | 0 (0) | 1 (4.5) |
| Aspirin alone | 1 (0.95) | 0 (0) | 0 (0) | 0 (0) | 1 (4.5) |
| **Antiplatelets and Anticoagulants** | 7 (6.7) | 2 (8.3) | 3 (6.3) | 0 (0) | 2 (9.1) |
| Warfarin | 3 (2.9) | 0 (0) | 1 (2.1) | 0 (0) | 2 (9.1) |
| NOAC | 4 (3.8) | 2 (8.3) | 2 (4.2) | 0 (0) | 0 (0) |
| Apixaban | 3 (2.9) | 2 (8.3) | 1 (2.1) | 0 (0) | 0 (0) |
| Rivaroxaban | 1 (0.95) | 0 (0) | 1 (2.1) | 0 (0) | 0 (0) |

Values are n (%).

NOAC: non-vitamin K oral anticoagulants.

**Supplementary Figure**

**Supplementary Figure 1. Quantitative coronary angiography showing percent diameter stenosis at baseline and after procedure for the specific treatment subgroups.**

The red line indicates the <20% residual stenosis threshold after procedure included in the primary effectiveness endpoint of the study.

B-IVL: bailout intravascular lithotripsy; DS: diameter stenosis; ISR: in-stent restenosis; ISR-IVL: intravascular lithotripsy for in-stent restenosis; IVL: intravascular lithotripsy; P-IVL: primary intravascular lithotripsy; QCA: quantitative coronary angiography; S-IVL: secondary intravascular lithotripsy.
