# Supplementary figures and images for: Intravascular Lithotripsy in Calcified Coronary Lesions: A Single-Center Experience in “Real-World” Patients
Source: Front Cardiovasc Med. 2022 Feb 21;9:829117. doi: 10.3389/fcvm.2022.829117 (PMC8900981; doi:10.3389/fcvm.2022.829117)

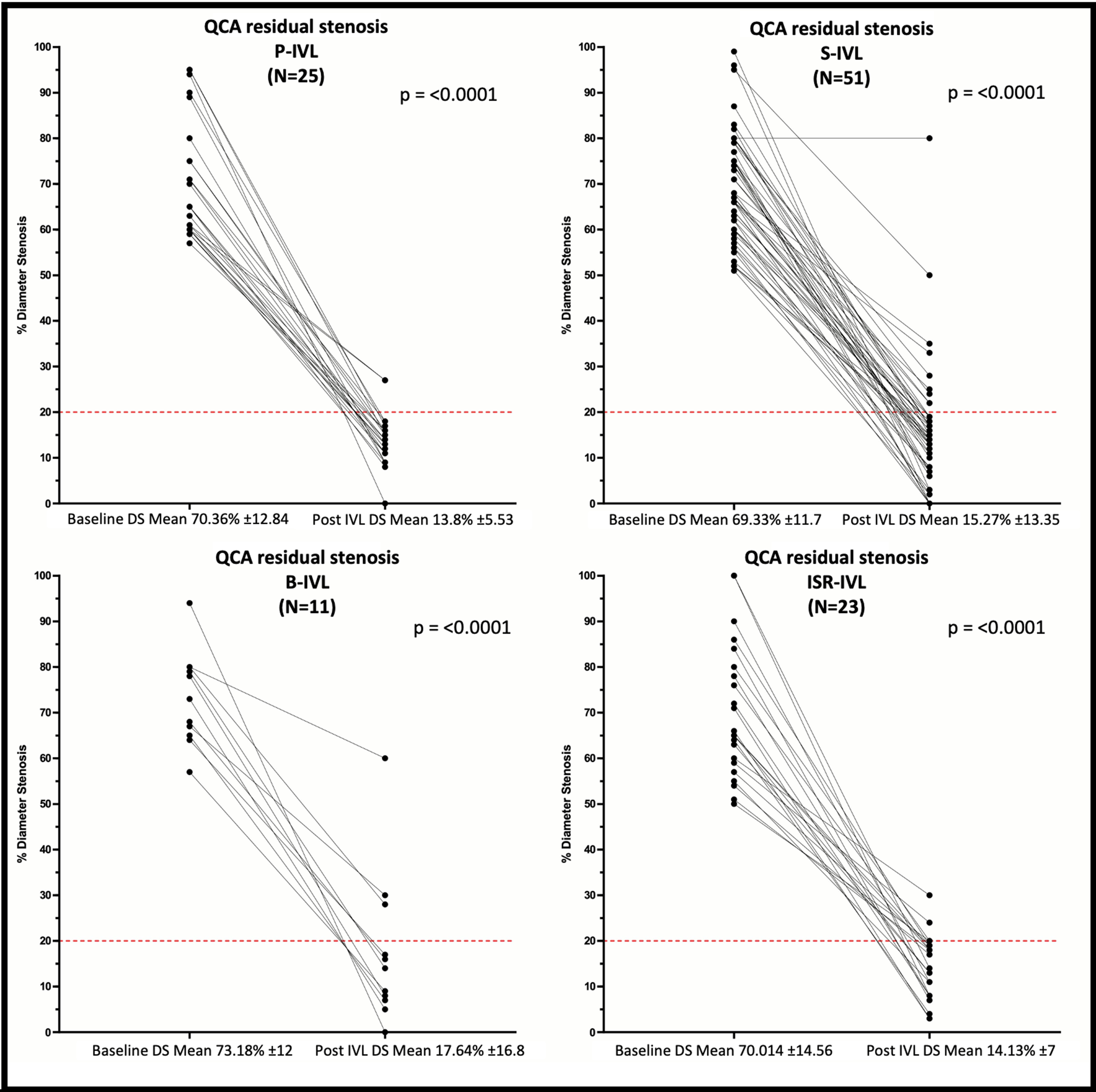

Supplement: Supplementary file 2 [file Image_2.TIFF]
